# Preferential Loss of Contrast Decrement Responses in Human Glaucoma

**DOI:** 10.1167/iovs.63.11.16

**Published:** 2022-10-20

**Authors:** Anthony M. Norcia, Alexandra Yakovleva, Naz Jehangir, Jeffrey L. Goldberg

**Affiliations:** 1 Department of Psychology, Wu Tsai Neurosciences Institute, Stanford University, Stanford, California, United States; 2 Spencer Center for Vision Research, Byers Eye Institute, Department of Ophthalmology, Stanford University, Stanford, California, United States

**Keywords:** glaucoma, ON/OFF pathways, luminance contrast, visual evoked potential (VEP)

## Abstract

**Purpose:**

The purpose of this study was to determine whether glaucoma in human patients produces preferential damage to OFF visual pathways, as suggested by animal experimental models, patient electroretinogram (ERG), and retinal imaging data.

**Methods:**

Steady-state visual evoked potentials (SSVEPs) were recorded monocularly from 50 patients with glaucoma and 28 age-similar controls in response to equal Weber contrast increments and decrements presented using 2.73 hertz (Hz) sawtooth temporal waveforms.

**Results:**

The eyes of patients with glaucoma were separated into mild (better than −6 decibel [dB] mean deviation; *n* = 28) and moderate to severe (worse than −6 dB mean deviation, *n* = 22) groups based on their Humphrey 24-2 visual field measurements. Response amplitudes and phases from the two glaucoma-severity groups were compared to controls at the group level. SSVEP amplitudes were depressed in both glaucoma groups, more so in the moderate to severe glaucoma group. The differences between controls and the moderate-severe glaucoma groups were more statistically reliable for decrements than for increments. Mean responses to decremental sawtooth stimuli were larger than those to increments in controls and in the mild glaucoma but not in the moderate to severe glaucoma group at the first harmonic. OFF/decrement responses at the first harmonic were faster in controls, but not in patients.

**Conclusions:**

The observed pattern of preferential loss of decremental responses in human glaucoma is consistent with prior reports of selective damage to OFF retinal ganglion cells in murine models and in data from human ERG and retinal imaging. These data motivate pursuit of SSVEP as a biomarker for glaucoma progression.

Glaucoma causes progressive damage to retinal ganglion cells (RGCs) and corresponding visual field losses.^1^ Treatment is only partially effective^2^^,^^3^ and thus a better understanding of the underlying pathophysiology is critical to the development of biomarkers for early detection or for progression, and to the development of new therapeutics. Recent work in murine models relevant to glaucoma has suggested preferential anatomic and functional damage occurs in OFF-center RGCs versus in ON-center RGCs.^4^^–^^9^ Although it is still unknown why and under what circumstances the structure and function of different RGC types may be preferentially affected by glaucoma,^10^ noninvasive assays of OFF versus ON pathway function may improve the detection of clinical glaucoma and progression monitoring if one pathway is preferentially affected.

Motivated by the findings in murine models.^4^^–^^9^ and results at the level of the retina from the electroretinogram (ERG) literature,^11^^,^^12^ here, we sought additional evidence for differential loss of OFF versus ON pathway function in patients with glaucoma. It is not possible to directly record the activity of OFF versus ON ganglion cells in humans, and therefore an indirect approach was taken using visual stimulation that is biased to activate either OFF- versus ON-derived pathways. Prior work in macaque monkeys has shown that ON ganglion cells are more responsive to sawtooth luminance stimuli in which the fast direction of the sawtooth results in a luminance increase. Similarly, OFF ganglion cells respond better to sawtooth stimulation where the fast direction creates a luminance decrement.^13^ Using sawtooth stimuli, we have found that sawtooth luminance stimulation generates differential steady-state visual evoked potentials (SSVEPs) to fast contrast increments versus decrements.^14^ These evoked responses had two features that parallel properties of ON and OFF pathways measured with single unit physiology in both cats and macaque monkeys. First, responses to contrast decrements were larger than responses to increments, consistent with a dark bias observed in single-cell responses in striate cortex of the cat^15^ and V1 of macaque monkeys^16^; see Ref. 17 for more extensive review and restrictions. Second, SSVEP responses to OFF-biased rapid decrements were faster than responses to increments as in cat LGN^18^ and striate cortex^19^ and in mouse retina.^20^

Here, we recorded SSVEPs to incremental and decremental sawtooth stimuli as a noninvasive reporter of the relative health of the underlying ON versus OFF pathway neurons of glaucoma patients. We find that SSVEPs to decremental/OFF stimulation are more affected than those to incremental/ON stimulation, particularly in patients with moderate to severe glaucoma.

## Methods

### Participants

Experiments proceeded after approval by the Institutional Review Board of Stanford University; written informed consent was obtained from all participants; all research conformed to the tenets of the Declaration of Helsinki in accord for use of human participants. All participants were employees of Stanford University or patients at the glaucoma and optometry clinics at the Byers Eye Institute at Stanford. For the patient groups, inclusion criteria included the diagnosis of glaucoma, best corrected visual acuity of 20/70 or better in the study eye(s), a mean deviation no worse than −25 decibels (dB) on the 24-2 Humphrey visual field (Zeiss Meditech, Dublin, CA, USA), absence of other ophthalmic issues that may impact vision, and cognitive abilities sufficient to participate in the study. Glaucoma was diagnosed based on glaucomatous optic nerve head damage on fundoscopic examination, including retinal nerve fiber layer thinning as measured on optical coherence tomography, and typical visual field loss on the 24-2 Humphrey visual field. This was defined as a positive glaucoma hemifield test or a cluster of at least 3 points below *P* = 0.05, with at least one point below *P* = 0.01. Humphrey visual field testing was performed for all the patients with glaucoma on the day of or within 3 months prior to the visual evoked potential (VEP) recording.

A group of 61 adults with glaucoma participated. Data from 11 patients were excluded based on one or more of the following criteria: extremely poor vision, electroencephalogram (EEG) data quality issues (excessive artifacts), corrupted data file or an incomplete recording. We report data from 50 eyes of 50 patients.

In addition, a group of 37 age- and gender-similar adults without glaucoma or other ocular pathologies and with normal visual acuity were recruited as controls. After the above exclusion criteria were applied, data from 28 controls were analyzed. Patient and control participant demographic data is summarized in Table 1.

**Table 1.** Participant Characteristics

	Patients		
	Mild Glaucoma	Moderate to Severe Glaucoma	Controls
	28 eyes	22 eyes	28 eyes (pooled)
Gender			
M	25		12
F	25		16
Age	61.71 y ± 13.3 (22–84 y)		62.4 y ±17.11 (18–80 y)
Race and ethnicity			
Caucasian	32		24
Asians	15		9
Hispanic	1		3
African American	1		–
Other	2		–
Diagnosis			–
Primary open angle glaucoma	15 eyes	18 eyes	
Juvenile open angle glaucoma	2 eyes	–	
Mixed mechanism	1 eye	–	
Normal tension glaucoma	6 eyes	2 eyes	
Pseudo exfoliation glaucoma	2 eyes	–	
Pigmentary dispersion glaucoma	1 eye	–	
Low tension	1 eye	2 eyes	
Mean deviation HVF	−3.17 ± 1.45	−12.78 ± 4.8	–
Mean BCVA LogMAR	+0.1± 0.13 (20/13-20/50 Snellen)	+0.1± 0.16 (20/13-20/70 Snellen)	0.016 ± 0.13 (20/10-20/60 Snellen)

For purposes of analysis, the eyes of the patients with glaucoma were split into two groups according to glaucoma severity based on a criterion of an eye having better (“mild”) or worse (“moderate to severe”) than a −6 dB mean deviation on Humphrey visual field testing. We analyzed one eye from a given patient, using the same eyes for both between-group comparisons and within-group comparison of the differences between ON/increment and OFF/decrement responses. Patients with only one eye meeting entry criteria were assigned to the severity group of that eye. For patients who had both eyes in the same severity group, or one eye mild and the other moderate to severe, the eye entered in the analysis was selected at random. Data from 28 eyes/patients with mild glaucoma and 22 eyes/patients with moderate to severe glaucoma were analyzed. The data from the two eyes of the control participants were averaged, yielding one estimate of the ON/increment and OFF/decrement response per control participant (*n* = 28 individuals).

### Visual Stimuli

**Figure 1.**
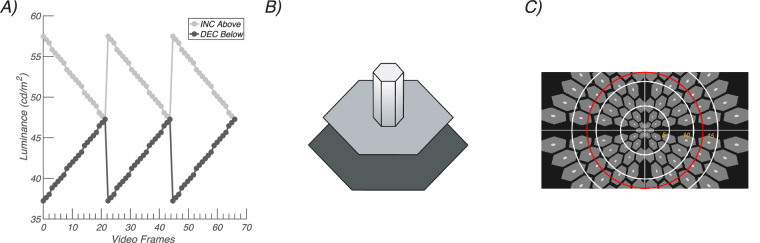
**(** **A**) Probe waveforms. Incremental (*gray*) and decremental (*black*) sawtooth waveforms designed to favor ON versus OFF pathway responses, respectively. The stimulus frequency of 2.73 Hz is illustrated. Frame rate was 60 Hz. (**B**) Probe-on-pedestal display element. The sawtooth-modulated probes (*small white hexagon*) were presented on a mid-gray pedestal (*medium size hexagon*). An incremental pedestal is illustrated. The probe was 20% the size of the pedestal. The pedestal was surrounded by a black background region (*largest hexagon*). Weber contrast was 20% for both increments and decrements. (**C**) Scaled stimulus array. The visual field was tiled with a set of probe/pedestal elements. The size of the elements was scaled over eccentricity according to the cortical magnification factor to optimize responses from the periphery. *White rings* indicate 5 degrees eccentricity radii from central fixation, and the *red ring* is 12 degrees in radius.

We used periodic decremental and incremental sawtooth stimulation to elicit SSVEPs.^14^ Decremental stimuli were defined as those in which the fast phase of the sawtooth decreases in luminance and the slow ramp phase increases (Fig. 1A, black curve), with incremental stimuli being the opposite (Fig. 1A, gray curve). The stimulus frequency was 2.73 hertz (Hz). Stimuli were presented on a SONY PVM-2541 monitor (1920 × 1080 pixels) viewed monocularly at a distance of 70 cm. Display pipeline delays and EEG pipeline delays were measured with a photocell and have been corrected.

The spatial structure of the stimulus was based on the Westheimer sensitization paradigm.^21^^,^^22^ In our version of the paradigm, small hexagonal probes were presented on larger hexagonal background pedestals that were five times larger than the probes (Fig. 1B). Pedestal luminance was 47 cd/m^2^, background luminance was 11 cd/m^2^, and probe contrast relative to the pedestal was +/−20% based on the Weber definition L_max_ − L_min_/L_min_. Increments and decrements had opposite signs but equal values in under this definition. The probes were modulated according to either a decremental or incremental sawtooth profile just described. The probes and pedestals were presented as multi-element arrays where each element comprised a probe-on-pedestal element. Elements that straddled the horizontal and vertical meridian were eliminated (see Fig. 1C for a schematic example). The probe and pedestal elements were scaled for cortical magnification as described previously.^14^ The central element comprised an 8 arc minute probe/40 arc minute pedestal. Field size was 12 degrees in radius vertically and approximately 20 degrees horizontally.

### Procedure

Participants viewed contrast increment and decrement stimuli monocularly with the other eye being patched. The trials (*n* = 10–12 per condition) were blocked by eye and within an eye-testing block, increment and decrement trials were presented in random order. Trials lasted 7.7–13.2 seconds with 3000 ± 500 msec inter-trial intervals. The first and last 1.1 seconds of each trial were excluded to allow for start-up and end effects. Participants were instructed to withhold blinking and to fixate on the center element. The participants performed a concurrent letter discrimination task presented within the central hexagon designed to control fixation and attention.

### SSVEP Recording and Signal Processing

The EEG was recorded over 128 channels using Hydrocell SensorNets and NetStation version 5.2 software (Electrical Geodesics, Eugene, OR, USA). Prior to recording, individual electrodes were adjusted so that the impedance values were lower than 60 kΩ. The raw EEG was amplified (gain = 1000 at 24-bit resolution) and digitally filtered with a zero-phase 0.3 Hz to 50 Hz bandpass filter. The data were then processed using in-laboratory software written in Objective C (XDiva). The artifact rejection procedure first detected and then substituted consistently noisy individual channels. The noisy channels were substituted with the average signals of the six nearest electrodes surrounding the noisy electrode. After this, the EEG was re-referenced to the common average of all electrodes. Second, to reject data recorded during coordinated muscle movements or blinks, 1-second-long epochs were excluded for all electrodes if signals of more than 5% (7 out of 128) of the electrodes exceeded a set threshold amplitude (60–520 µV, median = 100 µV) sometime during the epoch. Finally, 1 second epochs from individual electrodes were excluded if more than 10% of the epoch samples exceeded +/− 30 µV.

### Statistical Analysis

Reliable Components Analysis (RCA) was used to reduce the dimensionality of the 128 channel recordings to a small number of components.^23^ Each RCA component comprises a scalp topography and a response spectrum. RCA components were derived through an eigenvalue decomposition that maximized the trial-by-trial covariance matrix. This criterion reflects the fundamental assumption that the stimulus-driven evoked response is highly similar over repeated presentations of the same stimulus. RCA also results in an improved signal-to-noise ratio (SNR) and provides a data driven method for selecting the recording channels whose data are to be analyzed. Components were learned from the complex values of the first four harmonics of the stimulus frequency as higher harmonics had low SNR, even in control eyes.^14^ Spectral analysis was performed at the sensor level using a recursive least squares filter.^24^ The components were learned separately for the three groups pooling over stimulus polarity and eye. Each participant's complex-valued sensor-level data was projected through the group-specific weights and group averages were computed by coherent (vector) averaging of the complex values across trials and participants.

We used one-sample Hotelling's T^2^ or T^2^_circ_ tests to determine whether a response was present at a given harmonic, using the latter when its distribution assumptions were met and the former when they did not.^25^ We used two-sample T^2^ tests to compare ON/increments of OFF/decrement responses within participants and for assessing between group differences within a contrast polarity. Elliptical error bounds indicating +/−1 SEM were calculated as part of the T^2^ computation.^26^ Within each group, the difference between ON/increment- and OFF/decrement-averaged complex vectors were tested against a univariate null hypothesis of zero difference (test within subjects). Between groups, we used a multivariate test to determine if there was a significant difference between groups for ON/increment and OFF/decrement conditions separately (test between subjects). Hotelling's T^2^ test functions were written in MATLAB and results verified with R's Hotelling package (CRAN/Hotelling repository). Additional corrections to the standard calculations were made when the samples being compared had unequal covariance.^27^ Differences in correlations were determine with the cocor package in R (http://comparingcorrelations.org/). The presence of phase-angle differences between ON/increment and OFF/decrement responses was determined by via jackknifed estimates of the vector average phase differences between for control and patient groups.^28^^–^^30^

## Results

**Figure 2.**
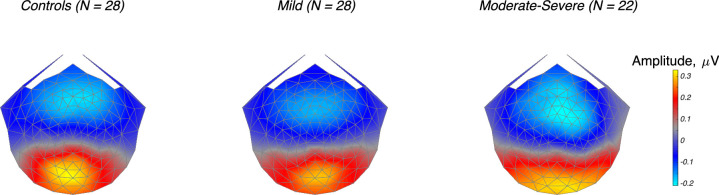
Response topography for the first RCA component (RC1) for controls, mild, and moderate to severe glaucoma groups. Responses over the first four harmonics of the stimulus frequency, 1F, 2F, 3F, and 4F are maximal over mid-line occipital electrodes. Color scale is the same for each group.

The SSVEP scalp topography of the dominant evoked response component, RC1 learned over both ON/increment and OFF/decrement conditions, was focused over the occipital pole in each of the participant groups, with the amplitude of the component decreasing with increasing glaucoma severity (Fig. 2).

### SSVEP Amplitude and Phase Versus Glaucoma Severity

**Figure 3.**
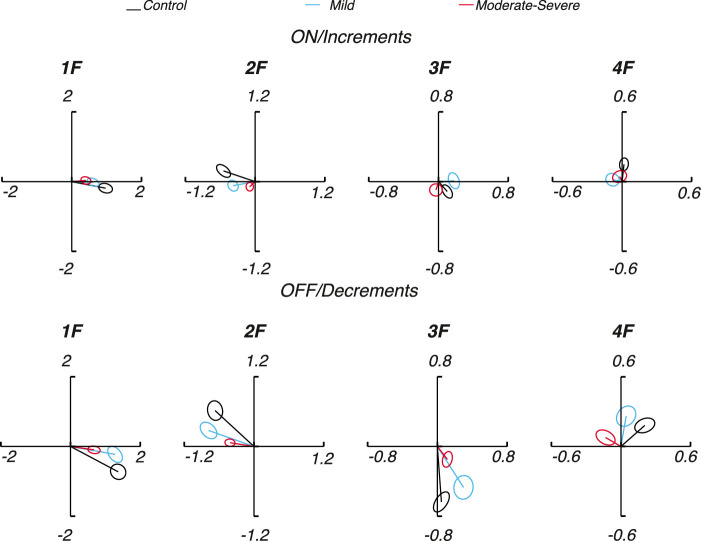
SSVEP 1F, 2F, 3F, and 4F amplitude and phase. *Top*: Responses to ON/increment sawtooth stimulation for control (*black*), mild (*cyan*), and moderate to severe (*red*) groups. *Bottom*: The same for OFF/decrement sawtooth stimulation. The phase origin is at 3 o'clock and increasing phase lag is in the counter-clockwise direction. Ellipses indicate the one standard error of the mean contour.

The SSVEP is complex-valued, comprising both amplitude and phase parameters at each response harmonic. For illustration of the complete spectral data set, Figure 3 shows the mean SSVEP responses for each group as a Nyquist plot where the x-axis represents the real (cosine) coefficient and the y-axis the imaginary (sine) coefficient, respectively. The distance from the origin of each vector represents the amplitude of SSVEP response; the angle represents the phase with increasingly delayed responses shifted counter-clockwise. The phase/time origin is at 3 o'clock. The ellipses represent the one standard error of the mean bound (see Methods).

Figure 3 top plots the complex amplitudes (1F, 2F, 3F, and 4F) for ON/increments and the bottom panel shows comparable data for OFF/decrements. In each panel, data from controls are shown in black, with data from mild and moderate to severe groups shown in cyan and red, respectively. Response amplitudes are generally lower in the patients than controls, especially for the moderate to severe group. Responses are overall delayed in the patients with glaucoma compared with controls, as indicated by the clockwise rotation of the phase angle of the response vectors away from the time origin (3 o'clock). For the 1F component, where significant responses are present for both ON/increment and OFF/decrement responses in all groups, the response is more delayed in the patients relative to the controls for OFF/decrements than for ON/increments (OFF = 18 vs. ON = 10 degrees for the mild group and OFF = 20 vs. ON = 16 degrees for the moderate to severe group). Responses are generally larger for OFF/decrement responses and both ON/increment and OFF/decrement responses are consistently smaller in the moderate to severe patient group than in the controls.

**Figure 4.**
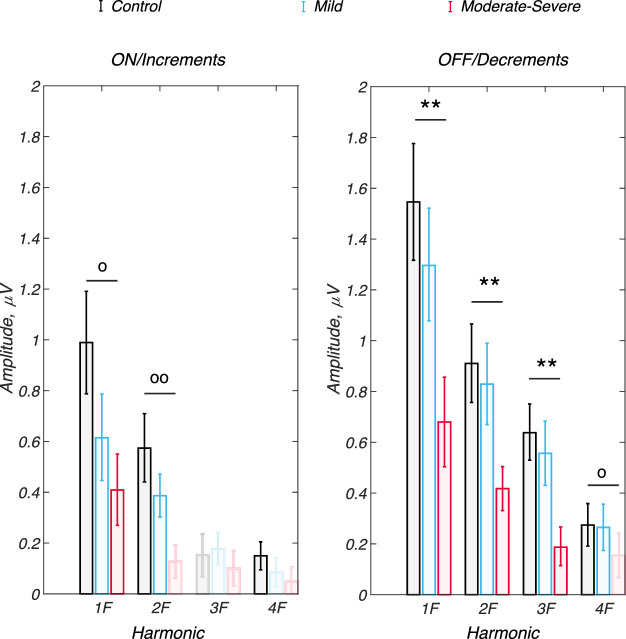
Amplitude versus harmonic number and pair-wise comparisons between groups for ON/increments (*left*) and OFF/decrements (*right*). The *P* values are derived from Hotelling's T^2^ statistic on the individual harmonic vector means. *P < 0.05, uncorrected. **P < 0.01, FDR corrected. Light shading indicates responses that are not measurably different than zero. See Table 2 for a complete set of comparisons.

To provide an alternative visualization of between group effects, Figure 4 plots the vector-mean amplitudes at each response harmonic for ON/increments (left) and OFF/decrements (right). Response amplitude generally decreases with glaucoma severity for both increments and decrements. Given that glaucoma results in a combination of amplitude and phase changes, we compared pairwise differences between groups using between-participant Hotelling's T^2^ tests that assess the joint effect of amplitude and phase differences. Significance values that survive the false discovery rate (FDR) cutoffs for the 12 between-group pairwise comparisons for ON and OFF responses are plotted above the vector mean amplitudes as stars. Those that are naively significant at the *P* < 0.05 level but do not survive FDR correction are plotted as open circles.

There are more cases of significant differences between patients and controls for decrements than increments. Four of 12 comparisons are naively significant at the *P* < 0.05, for decrements, whereas 2 reached the same naïve significance level for increments. Measurable differences after FDR thresholding of the naïve *P* values are present for controls versus patients with moderate to severe glaucoma at 1F, 2F, and 3F for OFF/decrements (see stars in Fig. 4, *P* = 0.004, *P* = 0.006, and *P* = 0.008, respectively). None of the ON/increment responses differences survived the FDR cutoff, although two comparisons were naively significant (2F, *P* = 0.005 and 1F, *P* = 0.024). The complete set of comparisons, with naïve *P* values and the FDR cutoffs are shown in Table 2.

**Table 2.** Pair-Wise Comparisons Between Groups for ON/Increment (Top) and OFF/Decrement Responses for 1f, 2F, 3F, and 4F Response Components

	ON/Increments			
Comparison	Rank	Naïve Significance	FDR Cutoff	FDR Significance
ModS_Cnt 2F	1	0.005	0.004	FALSE
ModS_Cnt 1F	2	0.024	0.008	FALSE
Mild_ModS 3F	3	0.056	0.013	FALSE
Mild_Cnt 4F	4	0.061	0.017	FALSE
Mild_ModS 2F	5	0.086	0.021	FALSE
Mild_Cnt 3F	6	0.133	0.025	FALSE
Mild_Cnt 2F	7	0.138	0.029	FALSE
Mild_Cnt 1F	8	0.158	0.033	FALSE
ModS_Cnt 4F	9	0.214	0.038	FALSE
ModS_Cnt 3F	10	0.581	0.042	FALSE
Mild_ModS 1F	11	0.626	0.046	FALSE
Mild_ModS 4F	12	0.881	0.050	FALSE
	**OFF/Decrements**			
**Comparison**	**Rank**	**Naïve Significance**	**FDR Cutoff**	**FDR Significance**

ModS_Cnt 1F	1	0.004	0.004	TRUE
ModS_Cnt 2F	2	0.006	0.008	TRUE
ModS_Cnt 3F	3	0.008	0.013	TRUE
ModS_Cnt 4F	4	0.032	0.017	FALSE
Mild_ModS 3F	5	0.078	0.021	FALSE
Mild_ModS 1F	6	0.096	0.025	FALSE
Mild_ModS 2F	7	0.099	0.029	FALSE
Mild_Cnt 1F	8	0.143	0.033	FALSE
Mild_ModS 4F	9	0.193	0.038	FALSE
Mild_Cnt 3F	10	0.243	0.042	FALSE
Mild_Cnt 2F	11	0.278	0.046	FALSE
Mild_Cnt 4F	12	0.488	0.050	FALSE

Cnt, controls; Mild, mild glaucoma group; ModS, moderate to –severe glaucoma group. Significance values for all possible pairwise comparisons are rank ordered (Rank) according to their T^2^ value (Naïve Significance). When naïve significance is better than FDR cutoff, significance after FDR correction is indicated as TRUE.

As just mentioned, differences between patient groups and controls measured by the T^2^ test reflect the combined effect of amplitude and phase differences. It is also of interest to ask to what extent individual patient VEP amplitudes correlated with MD, a common metric in the glaucoma literature. We computed Pearson correlation coefficients between mean deviation VEP amplitude at 1F, 2F, 3F, and 4F. Data were combined across the mild and the moderate to severe groups – *n* = 50. The correlations are significant for OFF/decrements and ON/increments for 1F (OFF-1F r = 0.39, *P* = 0.005 versus ON-1F r = 0.32, *P* = 0.02), and 2F (OFF-2F r = 0.38, *P* = 0.007 and ON-2F r = 0.28, *P* = 0.05). The level of these correlations, while numerically higher for OFF/decrements, do not differ between ON/increment and OFF decrement responses. None of the correlations were significant for 3F and 4F at the *P* < 0.05 level*.* Scatter plots are shown in Supplementary Figure S1.

### Partial Loss of OFF/Decrement Superiority in Moderate to Severe Glaucoma

**Figure 5.**
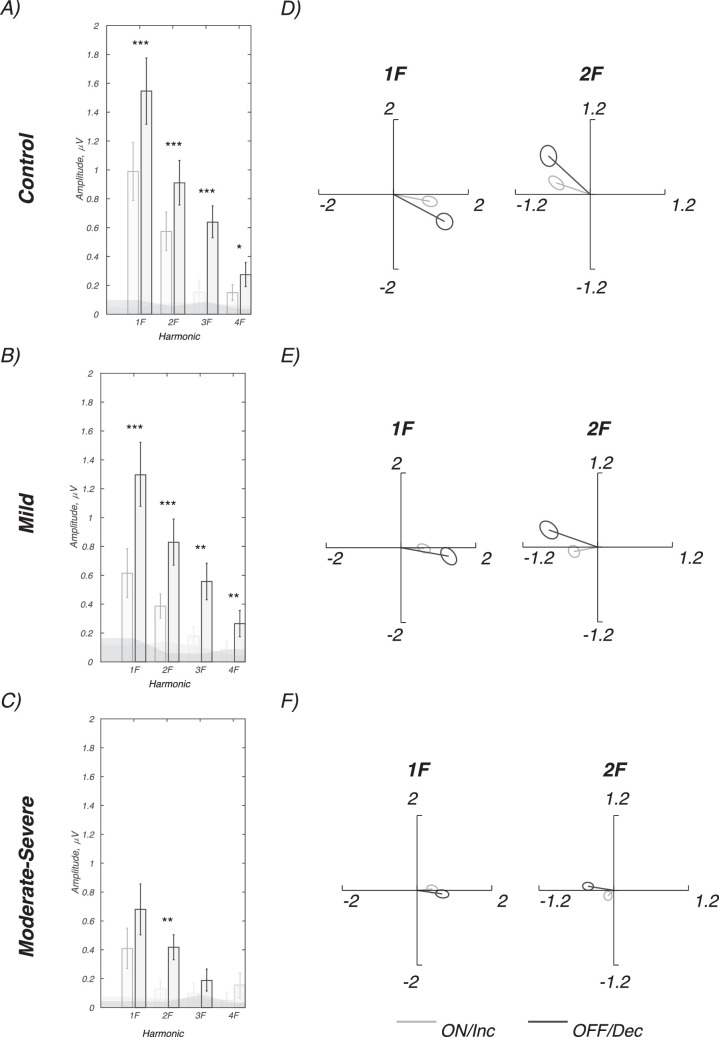
Amplitude versus harmonic number for ON/increments versus OFF/decrements for controls (**A**), mild (**B**), and moderate to severe glaucoma (**C**). Panels **D** to **F** plot 1F and 2F amplitude and phase for the same groups with 1 SEM error ellipses. Scale for real and imaginary components is microvolts. The *P* values in the *left panels* are derived from within-subjects Hotelling's T^2^ statistic on the harmonic vector means. *P < 0.05, **P < 0.01, ***P < 0.001, FDR corrected. *Gray* (ON/increment), and *black* (OFF/decrement) responses. Darker shading in **A** to **C** indicates statistically reliable response. See Table 2 for details of significance tests.

To better compare the effects of glaucoma severity on the functioning of ON versus OFF pathways, we made within-participant comparisons of ON/increment versus OFF/decrement responses using the same participants for mild (28 eyes) and for moderate to severe glaucoma groups (22 eyes) and controls (28 eyes). Figure 5 plots the vector mean amplitudes for 1F, 2F, 3F, and 4F for controls in panel A, the mild group in panel B, and the moderate to severe glaucoma group in panel C, and in polar format for 1F and 2F in panel B, the moderate to severe group in panel C, and the corresponding polar plots in panels D and E.

As reported previously for normal-vision adults,^14^ responses are larger for decrements than increments in the controls (19 of 28 control participants are common between studies). Responses in the control participants are also faster at 1F, and 2F for OFF/decrement stimulation as indicated by the phase advances. Patients with mild glaucoma also have significantly larger/faster decrement than increment responses for 1F and 2F. Patients with moderate to severe glaucoma have lower overall amplitudes than controls or patients with mild glaucoma (see also Fig. 4). Their responses to decrements are not measurably larger/different than those for increments at 1F where ON/increment and OFF/decrement responses are both significant. The lack of a difference in the moderate to severe glaucoma group at 1F is not a floor effect, as both responses are well above the noise level (gray bands) and are each significantly different from zero. Increment/decrement responses differ at 2F as they do in controls and patients with mild glaucoma, suggesting a partial sparing of function. The complete set of tests is listed in Table 3.

**Table 3.** Comparisons of ON/Increment versus OFF/Decrement Responses on a Within-Participant T^2^ Test for Control, Mild 3, and Moderate to Severe 3 Groups

	Control			
Comparison	Rank	Naïve Significance	FDR Cutoff	FDR Significance
Controls 3F	1	0.000	0.013	TRUE
Controls 2F	2	0.000	0.025	TRUE
Controls 1F	3	0.000	0.038	TRUE
Controls 4F	4	0.047	0.050	TRUE
	**Mild**			
**Comparison**	**Rank**	**Naïve Significance**	**FDR Cutoff**	**FDR Significance**
Mild 2F	1	0.000	0.013	TRUE
Mild 1F	2	0.000	0.025	TRUE
Mild 4F	3	0.005	0.038	TRUE
Mild 3F	4	0.005	0.050	TRUE
	**Moderate to Severe**			
**Comparison**	**Rank**	**Naïve Significance**	**FDR Cutoff**	**FDR Significance**
Moderate to severe 2F	1	0.003	0.013	TRUE
Moderate to severe 1F	2	0.116	0.025	FALSE
Moderate to severe 3F	3	0.495	0.038	FALSE
Moderate to severe 4F	4	0.546	0.050	FALSE

When naïve significance is better than FDR cut-off, significance after FDR correction is indicated as TRUE.

Evoked response shows a smaller phase lag with respect to the stimulus for OFF/decrement than ON/increment responses in healthy younger and older observers.^14^ Evoked response phase at 1F where both ON/increment and OFF/decrement responses are statistically reliable in each of the groups shows a smaller phase advance for OFF/decrements in the patient groups than in controls (10.1 and 9.2 degrees in mild and moderate to severe groups, respectively, versus 22.4 degrees in controls). This pattern comes about from the OFF/decrement responses being more delayed in patients than ON/increment responses (see discussion of Fig. 3). The phase difference between ON/increment and OFF/decrement responses are statistically reliable in the controls (*P* = 0.037), but not the mild *P* = 0.234 or moderate to severe patient groups (*P* = 0.368) on one-tailed *t*-tests. In the mild glaucoma group, a lack of significant difference is not due to a lack of response amplitude, as these are comparable to those of controls. This pattern is not present at 2F in the mild glaucoma group where both ON/increment and OFF/decrement responses are statistically reliable. Here, the OFF/decrement phase advantage is 29.8 degrees in the mild group versus 24 degrees in the controls.

## Discussion

Here, we find that SSVEP amplitudes are depressed and delayed in the moderate to severe glaucoma group, consistent with prior studies using VEP in patients with glaucoma.^31^ Importantly, these data expand upon several prior VEP studies by exploring responses to stimuli that favor the ON versus OFF visual pathways, showing that when considering the combination of amplitude and timing effects, differences between controls and patients with moderate to severe glaucoma are seen for more response components at higher levels of statistical reliability for decrements than for increments. In controls, the evoked response is significantly larger/faster for decrements than increments at all response harmonics. In patients with moderate to severe glaucoma, this enhanced OFF/decrement response is not present at 1F, is present at 2F, and is unmeasurable at 3F and 4F due to low SNR. In both patient groups, the phase advance of OFF/decrement versus ON/increment response is reduced relative to controls. Thus, when measured in this cohort trial design, there is preferential loss of responses that are likely to depend on the OFF pathway as glaucoma severity progresses. Losses first manifest in terms of response phase and then response amplitude as severity increases.

### Comparison With Animal Models of Glaucoma

Our results are consistent with evidence from animal models of glaucoma that have suggested earlier and/or more extensive damage to OFF RGCs than ON RGCs.^10^^,^^32^ After experimental elevation of IOP in mice, greater dendritic field shrinkage,^4^^–^^6^ greater reduction in receptive field size,^4^^,^^6^^,^^9^ greater cell death,^6^ and lower firing rates to visual stimulation have been reported in OFF versus ON RGCs. Other studies suggest that relative damage profiles in ON versus OFF RGCs may depend on the magnitude/temporal profile of IOP elevation or genetic background of the model,^33^^,^^34^ species,^35^ or method of quantifying receptive field size.^36^

Increased cell death in transient-OFF alpha RGCs has also been observed in a mouse optic nerve-crush model^8^ and other work using the optic nerve crush model has found that light responses are decreased earlier in OFF versus ON RGCs of mice, as are their receptive field sizes.^7^ Prolongation of response latency was also observed to occur earlier in OFF versus ON RGCs. Consistent with this, we find increased delays in patients versus controls that are larger for decrements than for increments. By contrast, another dataset comparing ON versus OFF RGCs in the mouse showed equal survival rates after axotomy, but ON-OFF types were more affected.^37^ At this point, it is premature to try to link the effects we see in the cortically derived SSVEPs to specific RGC pathophysiology. Differences measured here could be the result of a reduced number of functional OFF versus ON RGCs, reductions in their dendritic arbors or synaptic complements from other retinal neurons, changes in the strength of the drive that the remaining RGCs have on LGN or cortical processes, or a combination of these. Nevertheless, data from animal models at the level of the retina and RGCs support the hypothesis that the preferential loss of luminance decrement responses seen here in SSVEP is due to preferential loss of signaling originating at the level of the OFF RGCs in human patients as well.^38^

The current results provide further motivation to understand the biology of OFF and ON RGCs, because functional changes that may be related to changes seen in murine models can also be seen in human disease. These results motivate the further development of functional and structural assays of the two pathways, including ones other than the one we present here. Recent work with visible light optical coherence tomography,^39^ for example, has found evidence for thickness reductions in a portion of the inner plexiform layer containing both ON and OFF sub-laminae and suggestive evidence for thinning in an OFF-dominated sub-laminae but none in an ON-dominated sub-lamina. Our data provide an example of the SSVEP's ability to demonstrate this biological difference at the group level. This may be suitable for group level comparisons of different treatment regimes, or for comparisons between different types of optic neuropathy, as examples. Additional work will be needed to demonstrate SSVEP's suitability for use as a clinical diagnostic for individual patients.

### Comparison With Previous Behavioral Measurements in Patients With Glaucoma

Visual field defects are typically measured clinically with incremental stimuli, as in the Humphrey visual field and other common visual field analyzers. Decremental stimuli have been used for perimetry only rarely.^40^ Decremental perimetry compared favorably to conventional incremental perimetry (Humphry 24-2) in patients with glaucoma^41^ and was reported to detect field loss at locations with normal field sensitivity on incremental testing.^42^ A comparison of reaction times for incremental and decremental targets embedded in binary random noise found that reaction times were more elevated (slower) for increments than decrements in patients with glaucoma,^43^ suggesting that the ON pathway was more affected. Other work has reported that foveal increment and decrement thresholds are equally affected in glaucoma.^44^^,^^45^ However, when measurements were made at 9 degrees eccentricity, decremental thresholds were found to be elevated relative to increment thresholds, suggesting more OFF-pathway damage there, and pointing to retinal eccentricity of the targets as an important factor.^45^ The behavioral literature is thus mixed on whether the ON or OFF pathways are preferentially damaged in glaucoma, but our data detecting preferential loss of OFF pathway sensitivity adds to the preponderance of data on non-foveal vision, where glaucoma damage is more reliably detected in early and moderate disease. The next steps following from these studies could include additional dissection of the parafoveal and increasingly eccentric visual field to address these differences directly.

### Comparison With Previous VEP and ERG Studies in Patients With Glaucoma

Several previous studies have measured VEPs in patients with glaucoma using stimuli intended to bias functioning to ON versus OFF pathways. The first of these studies^46^ measured VEPs to sinusoidally modulated arrays of isolated bright or dark checks modulated at 10 Hz and presented on a mid-gray background. They reported that responses in the bright check condition were more affected in patients with glaucoma and glaucoma suspects. Subsequently, VEPs in patients with glaucoma and controls were measured for isolated checks of either 10% or 15% Weber contrast incremental and decremental stimuli.^47^ Using a criterion of whether a statistically reliable evoked response was present or not, receiver operating characteristic (ROC) analysis indicated that optimal increment stimuli demonstrated somewhat better classification rates than optimal decremental stimuli. Response magnitudes were not reported so it is difficult to compare their results to ours. Recent work with isolated checks has focused on responses to bright checks and has found these responses to be affected in glaucoma,^1^^,^^48^^–^^50^ but these data do not distinguish between ON versus OFF pathways.

Whether incremental or decremental SSVEP responses are seen to be more affected in glaucoma could depend on a variety of factors. The isolated check paradigm uses sinusoidal modulation, which is time-symmetric in terms of luminance increase/decrease. Sawtooth stimulation on the other hand is time-asymmetric and, along with its broad temporal frequency spectrum, this may more strongly differentially activate ON versus OFF pathways. Consistent with this, an ERG study using sawtooth stimulation found preferential loss of responses after the fast-phase of a sawtooth luminance modulation in glaucoma,^12^ extending prior work from the same group using long duration flashes to measure offset responses.^11^ Both studies found preferential loss of offset responses, consistent with our results.

Most studies of achromatic pattern evoked responses have found increased latency of transient evoked response peaks or delays in the steady state VEP,^51^^–^^60^ but some have found statistically reliable but small delays or delays that were not measurable.^46^^,^^61^^–^^64^ What we add to the previous studies is that in addition to overall delays in patients with glaucoma, the delay advantage for OFF/decrement responses in controls is lost in both mild and moderate-severe glaucoma due to a greater change in delay for responses to decrements.

A common feature of our study and previous VEP studies^46^^,^^47^^,^^50^^,^^65^ of increment/decrement sensitivity in glaucoma^9^^,^^40^^,^^43^^,^^44^ is that visual fields were defined using the Humphry 24-2 measurement that does not sample within the central +/− 3 degrees of the visual field. The VEP is dominated by the central visual field and, because of this, our stimulus arrays were scaled for cortical magnification. We have shown that scaling increases SSVEP amplitude,^14^ increasing the contribution to the overall VEP of peripheral locations where the visual field measurements are made. Eliciting a more robust response from the periphery may be important for detection ON/OFF differences as a previous psychophysical study in patients with glaucoma has found a greater deficit for detecting contrast decrements beyond the central 9 degrees.^45^ The use of multiple frequency stimulation^66^ to achieve analysis according to eccentricity may facilitate addressing this point, as discussed above. Taken together, the modifications in the current SSVEP protocol may more reliably differentiate between ON and OFF pathway responses, consistent with the larger effect of glaucoma on OFF pathways detected here.

## Conclusion

Our results suggest preferential damage to OFF-related visual pathways occurs in glaucoma. Given the diversity of previous findings in animal models and with behavioral measurements, further research is needed to understand the underlying biology of RGC susceptibility and to link this susceptibility to functional measurements. Future studies building on this foundational effort using longitudinal trial designs may reveal additional diagnostic utility (e.g. in predicting progression or response to therapy).

## Supplementary Material

Supplement 1

## References

[1] Quigley HA, Dunkelberger GR, Green WR. Retinal ganglion cell atrophy correlated with automated perimetry in human eyes with glaucoma. *Am J Ophthalmol*. 1989; 107: 453–464. 2712129 10.1016/0002-9394(89)90488-1

[2] Conlon R, Saheb H, Ahmed II. Glaucoma treatment trends: a review. *Can J Ophthalmol*. 2017; 52: 114–124. 28237137 10.1016/j.jcjo.2016.07.013

[3] Mathew DJ, Buys YM. Minimally Invasive Glaucoma Surgery: A Critical Appraisal of the Literature. *Annu Rev Vis Sci*. 2020; 6: 47–89. 32936738 10.1146/annurev-vision-121219-081737

[4] Della Santina L, Inman DM, Lupien CB, Horner PJ, Wong RO. Differential progression of structural and functional alterations in distinct retinal ganglion cell types in a mouse model of glaucoma. *J Neurosci*. 2013; 33: 17444–17457. 24174678 10.1523/JNEUROSCI.5461-12.2013 PMC3812509

[5] El-Danaf RN, Huberman AD. Characteristic patterns of dendritic remodeling in early-stage glaucoma: evidence from genetically identified retinal ganglion cell types. *J Neurosci*. 2015; 35: 2329–2343. 25673829 10.1523/JNEUROSCI.1419-14.2015 PMC6605614

[6] Ou Y, Jo RE, Ullian EM, Wong RO, Della Santina L. Selective Vulnerability of Specific Retinal Ganglion Cell Types and Synapses after Transient Ocular Hypertension. *J Neurosci*. 2016; 36: 9240–9252. 27581463 10.1523/JNEUROSCI.0940-16.2016 PMC5005727

[7] Puyang Z, Gong HQ, He SG, Troy JB, Liu X, Liang PJ. Different functional susceptibilities of mouse retinal ganglion cell subtypes to optic nerve crush injury. *Exp Eye Res*. 2017; 162: 97–103. 28629926 10.1016/j.exer.2017.06.014

[8] Daniel S, Clark AF, McDowell CM. Subtype-specific response of retinal ganglion cells to optic nerve crush. *Cell Death Discovery*. 2018; 5: 1–16. 10.1038/s41420-018-0069-y PMC6054657 30062056

[9] Sabharwal J, Seilheimer RL, Tao X, Cowan CS, Frankfort BJ, Wu SM. Elevated IOP alters the space-time profiles in the center and surround of both ON and OFF RGCs in mouse. *Proc Natl Acad Sci USA*. 2017; 114: 8859–8864. 28760976 10.1073/pnas.1706994114 PMC5565456

[10] Wang AY, Lee PY, Bui BV, et al. Potential mechanisms of retinal ganglion cell type-specific vulnerability in glaucoma. *Clin Exp Optom*. 2020; 103: 562–571. 31838755 10.1111/cxo.13031

[11] Horn FK, Gottschalk K, Mardin CY, Pangeni G, Junemann AG, Kremers J. On and off responses of the photopic fullfield ERG in normal subjects and glaucoma patients. *Doc Ophthalmol*. 2011; 122: 53–62. 21267627 10.1007/s10633-011-9258-1

[12] Pangeni G, Lammer R, Tornow RP, Horn FK, Kremers J. On- and off-response ERGs elicited by sawtooth stimuli in normal subjects and glaucoma patients. *Doc Ophthalmol*. 2012; 124: 237–248. 22457046 10.1007/s10633-012-9323-4

[13] Kremers J, Lee BB, Pokorny J, Smith VC. Responses of macaque ganglion cells and human observers to compound periodic waveforms. *Vision Res*. 1993; 33: 1997–2011. 8249315 10.1016/0042-6989(93)90023-p

[14] Norcia AM, Yakovleva A, Hung B, Goldberg JL. Dynamics of Contrast Decrement and Increment Responses in Human Visual Cortex. *Transl Vis Sci Technol*. 2020; 9: 6. 10.1167/tvst.9.10.6 PMC7476656 32953246

[15] Jin JZ, Weng C, Yeh CI, et al. On and off domains of geniculate afferents in cat primary visual cortex. *Nat Neurosci*. 2008; 11: 88–94. 18084287 10.1038/nn2029 PMC2556869

[16] Yeh CI, Xing D, Shapley RM. “Black” responses dominate macaque primary visual cortex v1. *J Neurosci*. 2009; 29: 11753–11760. 19776262 10.1523/JNEUROSCI.1991-09.2009 PMC2796834

[17] Jansen M, Jin J, Li X, et al. Cortical Balance Between ON and OFF Visual Responses Is Modulated by the Spatial Properties of the Visual Stimulus. *Cereb Cortex*. 2019; 29: 336–355. 30321290 10.1093/cercor/bhy221 PMC6294412

[18] Jin J, Wang Y, Lashgari R, Swadlow HA, Alonso JM. Faster thalamocortical processing for dark than light visual targets. *J Neurosci*. 2011; 31: 17471–17479. 22131408 10.1523/JNEUROSCI.2456-11.2011 PMC3470425

[19] Komban SJ, Kremkow J, Jin J, et al. Neuronal and perceptual differences in the temporal processing of darks and lights. *Neuron*. 2014; 82: 224–234. 24698277 10.1016/j.neuron.2014.02.020 PMC3980847

[20] Nichols Z, Nirenberg S, Victor J. Interacting linear and nonlinear characteristics produce population coding asymmetries between ON and OFF cells in the retina. *J Neurosci*. 2013; 33: 14958–14973. 24027295 10.1523/JNEUROSCI.1004-13.2013 PMC3771031

[21] Westheimer G. Spatial interaction in the human retina during scotopic vision. *J Physiol*. 1965; 181: 881–894. 5881260 10.1113/jphysiol.1965.sp007803 PMC1357689

[22] Westheimer G. Spatial interaction in human cone vision. *J Physiol*. 1967; 190: 139–154. 6038016 10.1113/jphysiol.1967.sp008198 PMC1365409

[23] Dmochowski JP, Greaves AS, Norcia AM. Maximally reliable spatial filtering of steady state visual evoked potentials. *Neuroimage*. 2015; 109: 63–72. 25579449 10.1016/j.neuroimage.2014.12.078 PMC6583904

[24] Tang Y, Norcia AM. An Adaptive Filter for Steady-State Evoked-Responses. *Evoked Potential*. 1995; 96: 268–277. 10.1016/0168-5597(94)00309-3 7750452

[25] Baker DH. Statistical analysis of periodic data in neuroscience. *ArXiv* *Preprints* 2021, 10.51628/001c.27680.

[26] Victor JD, Mast J. A new statistic for steady-state evoked potentials. *Electroencephalogr Clin Neurophysiol*. 1991; 78: 378–388. 1711456 10.1016/0013-4694(91)90099-p

[27] Kawasaki T, Seo T. A Two Sample Test for Mean Vectors with Unequal Covariance Matrices. *Commun Stat Simul Comput*. 2015; 44: 1850–1866.

[28] Miller RG. Jackknife - Review. *Biometrika*. 1974; 61: 1–15.

[29] Norcia AM, Tyler CW. Temporal Frequency Limits for Stereoscopic Apparent Motion Processes. *Vis Res*. 1984; 24: 395–401. 6740960 10.1016/0042-6989(84)90037-3

[30] Miller J, Patterson T, Ulrich R. Jackknife-based method for measuring LRP onset latency differences. *Psychophysiology*. 1998; 35: 99–115. 9499711

[31] Tai TYT. Visual Evoked Potentials and Glaucoma. *Asia Pac J Ophthalmol (Phila)*. 2018; 7: 352–355. 29638049 10.22608/APO.2017532

[32] Della Santina L, Ou Y. Who's lost first? Susceptibility of retinal ganglion cell types in experimental glaucoma. *Exp Eye Res*. 2017; 158: 43–50. 27319294 10.1016/j.exer.2016.06.006 PMC5161723

[33] Risner ML, Pasini S, Cooper ML, Lambert WS, Calkins DJ. Axogenic mechanism enhances retinal ganglion cell excitability during early progression in glaucoma. *Proc Natl Acad Sci USA*. 2018; 115: E2393–E2402. 29463759 10.1073/pnas.1714888115 PMC5877940

[34] Tao X, Sabharwal J, Seilheimer RL, Wu SM, Frankfort BJ. Mild Intraocular Pressure Elevation in Mice Reveals Distinct Retinal Ganglion Cell Functional Thresholds and Pressure-Dependent Properties. *J Neurosci*. 2019; 39: 1881–1891. 30622167 10.1523/JNEUROSCI.2085-18.2019 PMC6407300

[35] Zhou Y, Wang W, Ren B, Shou T. Receptive field properties of cat retinal ganglion cells during short-term IOP elevation. *Invest Ophthalmol Vis Sci*. 1994; 35: 2758–2764. 8188469

[36] Chen H, Zhao Y, Liu M, et al. Progressive degeneration of retinal and superior collicular functions in mice with sustained ocular hypertension. *Invest Ophthalmol Vis Sci*. 2015; 56: 1971–1984. 25722210 10.1167/iovs.14-15691 PMC4365983

[37] Tran NM, Shekhar K, Whitney IE, et al. Single-Cell Profiles of Retinal Ganglion Cells Differing in Resilience to Injury Reveal Neuroprotective Genes. *Neuron*. 2019; 104: 1039–1055.e1012. 31784286 10.1016/j.neuron.2019.11.006 PMC6923571

[38] Beykin G, Norcia AM, Srinivasan VJ, Dubra A, Goldberg JL. Discovery and clinical translation of novel glaucoma biomarkers. *Prog Retin Eye Res*. 2021; 80: 100875. 32659431 10.1016/j.preteyeres.2020.100875 PMC7796965

[39] Ghassabi Z, Kuranov RV, Schuman JS, et al. In Vivo Sublayer Analysis of Human Retinal Inner Plexiform Layer Obtained by Visible-Light Optical Coherence Tomography. *Invest Ophthalmol Vis Sci*. 2022; 63: 18. 10.1167/iovs.63.1.18 PMC8762683 35024761

[40] Mutlukan E, Damato BE. The dark perimetric stimulus. *Br J Ophthalmol*. 1992; 76: 264–267. 1390506 10.1136/bjo.76.5.264 PMC504252

[41] Mutlukan E, Damato BE, Jay JL. Clinical evaluation of a multi-fixation campimeter for the detection of glaucomatous visual field loss. *Br J Ophthalmol*. 1993; 77: 332–338. 8318478 10.1136/bjo.77.6.332 PMC504525

[42] Mutlukan E. Glaucomatous optic neuropathy causes sensitivity loss to light offsets in the visual field. *Neuroreport*. 1993; 4: 1159–1162. 8219010

[43] Zhao L, Sendek C, Davoodnia V, et al. Effect of Age and Glaucoma on the Detection of Darks and Lights. *Invest Ophthalmol Vis Sci*. 2015; 56: 7000–7006. 26513506 10.1167/iovs.15-16753 PMC4627468

[44] Sampson GP, Badcock DR, Walland MJ, McKendrick AM. Foveal contrast processing of increment and decrement targets is equivalently reduced in glaucoma. *Br J Ophthalmol*. 2008; 92: 1287–1292. 18614574 10.1136/bjo.2007.130880

[45] Joao CAR, Scanferla L, Jansonius NM. Retinal Contrast Gain Control and Temporal Modulation Sensitivity Across the Visual Field in Glaucoma at Photopic and Mesopic Light Conditions. *Invest Ophthalmol Vis Sci*. 2019; 60: 4270–4276. 31618763 10.1167/iovs.19-27123

[46] Greenstein VC, Seliger S, Zemon V, Ritch R. Visual evoked potential assessment of the effects of glaucoma on visual subsystems. *Vision Res*. 1998; 38: 1901–1911. 9797966 10.1016/s0042-6989(97)00348-9

[47] Zemon V, Tsai JC, Forbes M, et al. Novel electrophysiological instrument for rapid and objective assessment of magnocellular deficits associated with glaucoma. *Doc Ophthalmol*. 2008; 117: 233–243. 18483820 10.1007/s10633-008-9129-6

[48] Chen X, Zhao Y. Diagnostic performance of isolated-check visual evoked potential versus retinal ganglion cell-inner plexiform layer analysis in early primary open-angle glaucoma. *BMC Ophthalmol*. 2017; 17: 77. 28532392 10.1186/s12886-017-0472-9 PMC5440894

[49] Chen XW, Zhao YX. Comparison of isolated-check visual evoked potential and standard automated perimetry in early glaucoma and high-risk ocular hypertension. *Int J Ophthalmol*. 2017; 10: 599–604. 28503434 10.18240/ijo.2017.04.16 PMC5406639

[50] Xu LJ, Li SL, Zemon V, Xie YQ, Liang YB. Central visual function and inner retinal structure in primary open-angle glaucoma. *J Zhejiang Univ Sci B*. 2020; 21: 305–314. 32253840 10.1631/jzus.B1900506 PMC7183442

[51] Atkin A, Bodis-Wollner I, Podos SM, Wolkstein M, Mylin L, Nitzberg S. Flicker threshold and pattern VEP latency in ocular hypertension and glaucoma. *Invest Ophthalmol Vis Sci*. 1983; 24: 1524–1528. 6642932

[52] Towle VL, Moskowitz A, Sokol S, Schwartz B. The visual evoked potential in glaucoma and ocular hypertension: effects of check size, field size, and stimulation rate. *Invest Ophthalmol Vis Sci*. 1983; 24: 175–183. 6826322

[53] Price MJ, Drance SM, Price M, Schulzer M, Douglas GR, Tansley B. The pattern electroretinogram and visual-evoked potential in glaucoma. *Graefes Arch Clin Exp Ophthalmol*. 1988; 226: 542–547. 3209081 10.1007/BF02169202

[54] Parisi V, Miglior S, Manni G, Centofanti M, Bucci MG. Clinical ability of pattern electroretinograms and visual evoked potentials in detecting visual dysfunction in ocular hypertension and glaucoma. *Ophthalmology*. 2006; 113: 216–228. 16406535 10.1016/j.ophtha.2005.10.044

[55] Mokbel T, Ghanem A. Diagnostic Value of Color Doppler Imaging and Pattern Visual Evoked Potential in Primary Open-Angle Glaucoma. *J Clinic Experiment Ophthalmol*. 2011; 2: 127.

[56] Pillai C, Ritch R, Derr P, et al. Sensitivity and specificity of short-duration transient visual evoked potentials (SD-tVEP) in discriminating normal from glaucomatous eyes. *Invest Ophthalmol Vis Sci*. 2013; 54: 2847–2852. 23513061 10.1167/iovs.12-10097

[57] Kothari R, Singh R, Singh S, Bokariya P. Association of pattern reversal VEP parameters with the mean defect of Humphrey visual field in patients of primary open angle glaucoma. *Indian J Physiol Pharmacol*. 2013; 57: 123–131. 24617161

[58] Sponsel WE, Johnson SL, Trevino R, et al. Pattern Electroretinography and Visual Evoked Potentials Provide Clinical Evidence of CNS Modulation of High- and Low-Contrast VEP Latency in Glaucoma. *Transl Vis Sci Technol*. 2017; 6: 6. 10.1167/tvst.6.6.6 PMC5678951 29134137

[59] Jha MK, Thakur D, Limbu N, Badhu BP, Paudel BH. Visual Evoked Potentials in Primary Open Angle Glaucoma. *J Neurodegener Dis*. 2017; 2017: 9540609. 28808597 10.1155/2017/9540609 PMC5541795

[60] Baisakhiya S, Lal Goyal G, Garg P, Khan Z. Correlation Between VEP Latency, CDR and PSD on Standard Automated Perimetry in Newly Diagnosed POAG Cases. *Delhi J Ophthalmol*. 2018; 28-22: 19.

[61] Marx MS, Bodis-Wollner I, Lustgarten JS, Podos SM. Electrophysiological evidence that early glaucoma affects foveal vision. *Doc Ophthalmol*. 1987; 67: 281–301. 3447853 10.1007/BF00144282

[62] Porciatti V, Di Bartolo E, Nardi N, Fiorentini A. Responses to chromatic and luminance contrast in glaucoma: a psychophysical and electrophysiological study. *Vision Res*. 1997; 37: 1975–1987. 9274782 10.1016/s0042-6989(97)00018-7

[63] Rodarte C, Hood DC, Yang EB, et al. The effects of glaucoma on the latency of the multifocal visual evoked potential. *Br J Ophthalmol*. 2006; 90: 1132–1136. 16707520 10.1136/bjo.2006.095158 PMC1857385

[64] Grippo TM, Hood DC, Kanadani FN, et al. A comparison between multifocal and conventional VEP latency changes secondary to glaucomatous damage. *Invest Ophthalmol Vis Sci*. 2006; 47: 5331–5336. 17122121 10.1167/iovs.06-0527

[65] Xu LJ, Zhang L, Li SL, Zemon V, Virgili G, Liang YB. Accuracy of isolated-check visual evoked potential technique for diagnosing primary open-angle glaucoma. *Doc Ophthalmol*. 2017; 135: 107–119. 28702796 10.1007/s10633-017-9598-6

[66] Nakanishi M, Wang YT, Jung TP, et al. Detecting Glaucoma With a Portable Brain-Computer Interface for Objective Assessment of Visual Function Loss. *JAMA Ophthalmol*. 2017; 135: 550–557. 28448641 10.1001/jamaophthalmol.2017.0738 PMC5772598

