# Supplementary figures and images for: Preferential Loss of Contrast Decrement Responses in Human Glaucoma
Source: Invest Ophthalmol Vis Sci. 2022 Oct 20;63(11):16. doi: 10.1167/iovs.63.11.16 (PMC9587510; doi:10.1167/iovs.63.11.16)

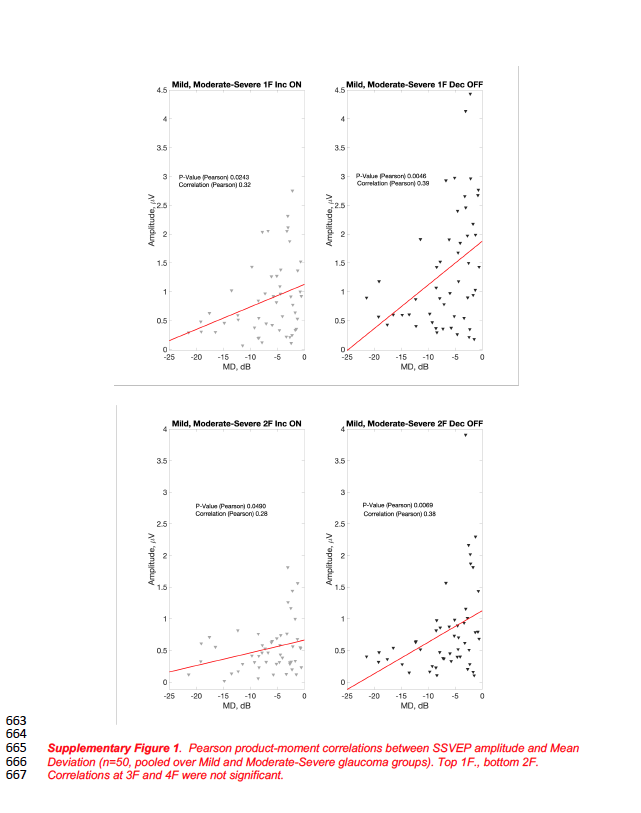

Supplement: Supplement 1 [file iovs-63-11-16_s001.png]
